# Changes in information-seeking patterns and perception of health crisis management in a year of COVID-19 pandemic: a repeated cross-sectional study

**DOI:** 10.3325/cmj.2023.64.93

**Published:** 2023-04

**Authors:** Aleksandra Banić, Damir Sapunar, Ivan Buljan

**Affiliations:** 1 University of Split, School of Medicine, Split, Croatia; 2 Laboratory for Pain Research, University of Split, School of Medicine, Split, Croatia; 3 Centre for Evidence-Based Medicine and Health Care, Catholic University of Croatia, Zagreb, Croatia; 4 Department of Research in Biomedicine and Health, University of Split, School of Medicine, Split, Croatia; 5 Department of Psychology, Faculty of Humanities and Social Sciences in Split, University of Split, Split, Croatia

## Abstract

**Aim:**

To assess the changes in the health information search patterns related to the COVID-19 pandemic and the use of this information in Croatia.

**Methods:**

This repeated cross-sectional study was based on an online survey among adults in Croatia from June 5 to July 5 2020 and from May 25 to June 15 2021. The survey inquired about demographic characteristics, health information search patterns, and emotional reactions to health information. The differences between the year 2020 and the year 2021 were assessed.

**Results:**

The survey was completed by 569 respondents (median age 38.5 years) in 2020 and by 598 respondents (median age 40 years) in 2021. In 2020, institutional governmental bodies were perceived to be a reliable source of information, but this perception declined in 2021. Whereas in 2020 TV was the most used source of health-related information, online media prevailed in 2021. After one year of the pandemic, respondents attributed significantly greater importance to the reliability of the information obtained from different sources.

**Conclusion:**

Our results may be valuable in designing public health communication strategies and campaigns, in selecting communication channels and sources, and in tailoring health information according to the characteristics and habits of the studied population.

During times of crisis (1), people need access to accurate information (2). According to the World Health Organization, in the first months of the COVID-19 pandemic we were struggling not only with a pandemic but also with an infodemic (3,4), with fake news and misinformation spreading through the internet. The media, science, and health literacy (5,6) play a major role in distinguishing between reliable information and misinformation on COVID-19 (7,8).

Before the first COVID-19 case was reported in Croatia (9), the Croatian government established the Civil Protection Headquarters (CPH), an expert group for pandemic management (10). The CPH included 26 representatives from hospitals, ministries, civil services, and governmental institutions (11). The Government also launched an official website, where it published all the information related to COVID-19 (12). It also used social media platforms, such as Facebook, Twitter, Instagram, and YouTube, to provide timely and accurate pandemic-related information.

The CPH was meeting on a regular basis and informing the public through press conferences. The adopted approach was in line with lessons learned from previous public health crises (13,14) and recommendations from reliable and trusted sources of communication (15). Nevertheless, the CPH was a subject of occasional criticism, and the media reported on the personal and professional oversights of its members (16,17).

The media coverage of the pandemic frequently involved individuals from the scientific community, medical profession, and pseudoscientific circles commenting on various COVID-19-related issues (18-27). While appropriate communication through the media can improve citizens’ knowledge, media literacy, and compliance with epidemiological measures (28-31), frequent exposure to news and social media may result in information overload (32), anxiety or fear (33-35), and depression (36,37).

Numerous studies in 2020 investigated the sources of information and search patterns related to the COVID-19 pandemic (38-41). However, no studies investigated whether the information search patterns changed as the pandemic progressed. The aim of this study was to investigate the change in search patterns and use of health information related to COVID-19 in Croatia three months after the outbreak and one year later. We hypothesized that the search patterns and information use changed, and that the trust in CPH declined after a year of the pandemic. Therefore, we identified information sources, information channels, search frequency, trust in the sources, and emotions provoked by consuming information related to COVID-19.

## Participants and methods

### Study design, setting, and participants

In this repeated cross-sectional study, we collected data from June 5 to July 5, 2020 and from May 25 to June 15, 2021. The survey was distributed to disease-specific patient associations (42), whose members were patients, people without diseases, non-expert population, and health care professionals. The contacts of these associations were freely available. We encouraged association members and all other participants to distribute the survey link further. This study is reported as per the Strengthening the Reporting of Observational Studies in Epidemiology guidelines (43). It was approved by the Ethics Committee of the Split School of Medicine.

### Procedure

The study instrument (Supplemental Material 1(Supplementary material 1)) was developed by using an online survey system (www.surveymonkey.com). In both years, the questionnaire had an identical structure.

The only inclusion criterion was the age of 18 years and over, while there were no exclusion criteria. Before filling out the survey, the respondents were informed about the survey purpose, and all respondents provided informed consent.

### Outcomes

We collected information on sex, age, education level, employment status, and whether the respondents worked in a medical profession. Information on respondents’ health status, chronic diseases in particular, was also collected to assess the number of people in each year who had specific health issues. We used this variable to avoid bias, since we assumed that healthy respondents would be less vulnerable to COVID-19 and thus less attentive to health information.

The respondents rated their concern for personal health during the pandemic on a scale from 1 (strongly disagree) to 5 (strongly agree). They also noted their most frequent source of information during the pandemic (TV, radio, online media, or printed media), aspects of its use, the need to seek additional information, and search frequency. The respondents rated the quality and clarity of information, the benefits from information provided by the CPH and the media, and its usefulness and reliability.

The respondents rated the reliability and trustworthiness of individual public figures as sources of information related to the COVID-19 pandemic on a scale from 1 (completely unreliable) to 5 (absolutely reliable). The public figures were grouped in four categories: health professionals, scientists who frequently appeared in the media, media-exposed politicians, and public figures who promoted anti-vaccination theories and non-conventional and alternative approaches to pandemic issues.

From a list of emotions, the respondents were asked to identify an emotion that they felt when they were exposed to pandemic-related information from online sources.

### Validation procedure

We first assessed the face-validity of the instrument, with all research team members examining the items for clarity and relevance to the COVID-19 behaviors and opinions (44,45). In the second step, we conducted content validity assessment by sending the survey to a small number of respondents to confirm its fluency (46). After verifying that participants had answered to all questions, we continued data collection.

### Study size

In line with another study that investigated knowledge, attitudes, and practices related to COVID-19 (47), we assumed that individuals from 2020 would be more concerned for their health, so we hypothesized that at least 28.4% of respondents in 2021 and 43.6% of respondents in 2020 would be concerned for their health due to the COVID-19 pandemic. With 80% power and an alpha error of 0.05, we needed a minimum of 153 participants per group to observe a meaningful difference (48).

### Statistical analysis

Categorical variables are presented as frequencies and percentages, while numerical variables are presented as means with 95% confidence intervals. The significance of differences between 2020 and 2021 was assessed with a χ^2^ test for categorical variables and a *t* test for continuous variables. Where necessary, we adjusted the probability values by applying the Bonferroni correction (0.05/22 = 0.002). All the analyses were conducted with JASP software, version 0.14.1.0 (JASP Team, 2020).

## Results

The survey was completed by 569 respondents (median age: 38.5 years) in 2020 and by 598 respondents (median age: 40 years) in 2021. In both years, the majority of respondents were women and respondents with a higher educational degree and employment rate. Less than half of the respondents were concerned for their health (45.8% in 2020, 42.2% in 2021), but significantly more respondent in 2021 than in 2020 believed that they would get infected with COVID-19 (*P* < 0.001) (Table 1).

**Table 1:** Demographic data and personal health-related variables

Variable	2020 (N = 569)	2021 (N = 598)	P*
Sex, n (%)^†^			
male	140 (24.6)	164 (27.7)	0.462
female	425 (74.7)	426 (71.8)	
does not state	4 (0.7)	3 (0.5)	
Age (mean, 95% CI)	38.5 (37.5-40.0)	40 (39.1-40.0)	0.018
Education degree, n (%)^‡^			
elementary school	4 (0.7)	2 (0.3)	0.762
high school	171 (30.9)	174 (29.5)	
bachelor	86 (15.6)	84 (14.3)	
college	229 (41.4)	254 (43.1)	
master’s degree	25 (4.5)	35 (5.9)	
PhD	38 (6.9)	40 (6.8)	
Employment status, n (%)^‖^			
employed	395 (69.5)	448 (75.8)	0.038
unemployed	53 (9.3)	48 (8.1)	
student	77 (13.6)	53 (9.0)	
retired	27 (4.8)	33 (5.6)	
other	16 (2.8)	9 (1.5)	
Physician or other health care worker, n (%)	66 (11.6)	78 (13.1)	0.422
Type of chronic disease, n (%)			
pulmonary diseases	12 (2.1)	7 (1.7)	0.161
asthma	12 (2.1)	9 (1.5)	
cardiovascular diseases	26 (4.6)	28 (4.6)	
diabetes	13 (2.3)	15 (2.5)	
immunological disease	62 (10.9)	34 (5.6)	
kidney disease	1 (0.2)	0 (0.0)	
overweight	33 (5.8)	24 (4.0)	
liver diseases	9 (1.6)	1 (0.2)	
The assessment of current health condition (1-5, median, 95% CI)	4.1 (4.0-4.1)	4.2 (4.1-4.3)	0.006
Were you more concerned for your health during the COVID-19 pandemic than usual? (Yes), n (%)^§^	260 (45.8)	251 (42.2)	0.218
COVID-19 presents a serious health issue for me. (1-5, median, 95% CI)	3.1 (3.0-3.2)	3.0 (2.9-3.1)	0.131
I am afraid that I could get infected with COVID-19. (1-5, median, 95% CI)	3.0 (2.9-3.1)	2.8 (2.7-2.9)	0.025
I will probably get infected with COVID-19 and get sick. (1-5, median, 95% CI)	2.4 (2.3-2.4)	2.9 (2.8-2.9)	**<0.001**
I feel that the infection would be very dangerous for me. (1-5, median, 95% CI)	2.6 (2.5-2.6)	2.4 (2.3-2.5)	0.007

*Chi squared for categorical variables and *t* test for continuous variables; Bonferroni correction 0.05/12 = 0.004.

†Four answers missing for 2021.

‡Sixteen answers missing for 2020 and nine for 2021.

‖One answer missing for 2020 and seven for 2021.

§One answer missing for 2021.

The number of respondents who relied on information from the CPH significantly declined in 2021 compared with 2020, and respondents switched to online media or avoided the media altogether. Respondents in 2021 also reported less frequent information search, and perceived reporting on COVID-19 as politically biased. Furthermore, they evaluated information on COVID-19 more critically compared with the respondents from 2020 (Table 2).

**Table 2:** Health information-seeking characteristics during the COVID-19 pandemic

Variable	2020 (N = 569)	2021 (N = 598)	P*
During the COVID 19 pandemic I mostly relied on information provided by Civil Protection Headquarters (Yes), n (%)^†^	434 (76.3)	251 (42.3)	**<0.001**
The media I most frequently used, n (%)^‡^			
television	249 (43.9)	129 (21.5)	**<0.001**
radio	7 (1.2)	4 (0.7)	
online media	270 (47.6)	367 (60.4)	
printed media	5 (0.9)	6 (1.0)	
I did not follow media	36 (6.4)	92 (15.4)	
I had the urge to seek additional information about COVID-19 online (Yes), n (%)^‡^	326 (57.3)	389 (65.2)	0.006
I had the urge seek additional information about COVID-19, n (%)^§^			
less than once a day	187 (33.0)	327 (54.8)	**<0.001**
only once a day	193 (34.1)	149 (25.0)	
two times a day	101 (17.5)	74 (12.4)	
three or more times a day	85 (15.0)	46 (7.8)	
Information provided by the Civil Protection Headquarters was clear and useful. (Mean, 95% CI)	3.6 (3.5-3.7)	2.6 (2.5-2.7)	**<0.001**
The Civil Protection Headquarters consists of professionals who know what they are doing, and act based on knowledge. (Mean, 95% CI)	3.5 (3.5-3.7)	2.7 (2.6-2.8)	**<0.001**
Information given by the Civil Protection Headquarters is a result of politics and not medical profession. (Mean, 95% CI)	2.8 (2.7-2.9)	3.5 (3.4-3.6)	**<0.001**
Articles on the pandemic in daily print are sensationalistic. (Mean, 95% CI)	3.6 (3.5-3.7)	3.8 (3.7-3.9)	**<0.001**
Journalists are well informed on the pandemic. (Mean, 95% CI)	2.5 (2.5-2.6)	2.3 (2.2-2.4)	**<0.001**
Newspaper articles on the pandemic are politically biased. (Mean, 95% CI)	3.2 (3.2-3.3)	3.6 (3.5-3.7)	**<0.001**
Information on the pandemic acquired online is confusing and often contradictory. (Mean, 95% CI)	3.5 (3.4-3.6)	3.6 (3.5-3.7)	0.012
Information on the pandemic acquired online is useful when we want-be better informed. (Mean, 95% CI)	3.3 (3.3-3.4)	3.4 (3.3-3.5)	0.299
When searching information online one should watch for accuracy and reliability of information. (Mean, 95% CI)	4.3 (4.2-4.3)	4.2 (4.1-4.3)	0.621
When I read information in the media about COVID-19 I wonder (Mean, 95% CI):			
...if the information is under political influence.	3.3 (3.2-3.4)	3.7 (3.6-3.8)	**<0.001**
…if the content is scientifically or professionally sound.	3.5 (3.4-3.6)	3.9 (3.8-4.0)	**<0.001**
…whether the source is trustworthy of fake.	3.7 (3.6-3.8)	4.0 (3.9-4.1)	**<0.001**
…if the information is up to date or outdated.	3.4 (3.4-3.5)	3.7 (3.6-3.8)	**<0.001**
…whether the source is independent, or the data depends on other sources.	3.4 (3.3-3.5)	3.8 (3.7-3.8)	**<0.001**
…if the information collected from the source is biased.	3.4 (3.3-3.5)	3.8 (3.7-3.9)	**<0.001**
…if there is contradictory information.	3.4 (3.3-3.5)	3.7 (3.6-3.8)	**<0.001**

*Chi squared for categorical variables and *t* test for continuous variables, Bonferroni correction 0.05/20 = 0.0025.

†One answer missing for 2021.

‡Two answers missing for 2020.

§Three answers missing for 2020 and two for 2021.

**Figure 1:**
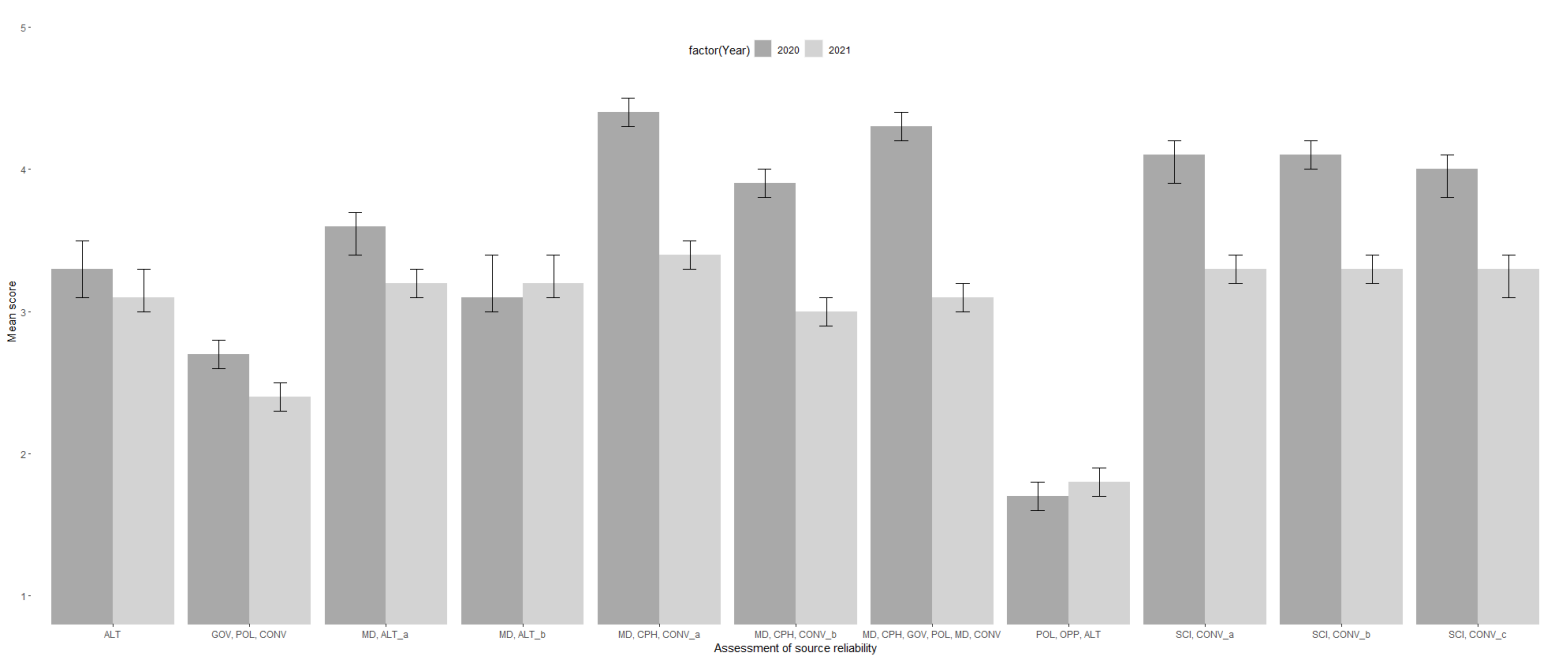
Reliability assessment of media-exposed figures (mean, 95% confidence interval) in 2020 (dark gray) and 2021 (light gray), on a scale from 1 (completely unreliable) to 5 (absolutely reliable). Personal names were replaced with the figure’s respective field of public activity relevant for the research, as follows: GOV – an individual was part of the government, a member of the political party with a majority in parliament; SCI – a scientist; MD – a medical doctor; CPH – a member of the Civil Protection Headquarters; POL – a politician; OPP – a member of a parliamentary opposition party; CONV – a person who promoted conventional attitudes toward medicine and public health; ALT – a person who promoted alternative, pseudo-, or non-scientific attitudes toward medicine and public health. One public figure may be assigned several codes.

In 2021, almost all categories of public figures were perceived as less reliable (*P* < 0.001), except the figures who argued against conventional medicine and COVID-19 protective measures (Figure 1). The number of respondents who were not familiar with the figures promoting pseudoscience slightly decreased in 2021. Still, respondents were less familiar with figures promoting alternative approaches than with politicians or medical experts (Supplemental Figure 1(Supplementary Figure 1)).

**Figure 2:**
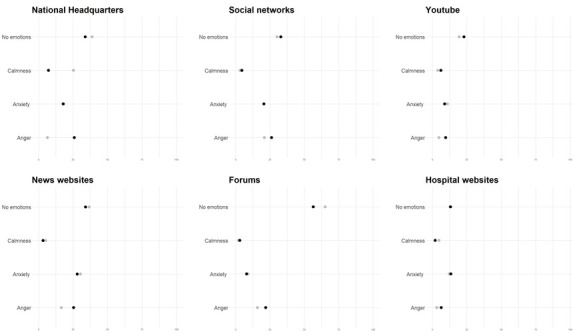
The proportion of participants in 2020 (black dot) and 2021 (gray dot) who experienced a particular emotion provoked by a different online source.

In most respondents in 2020 and 2021, information search related to COVID-19 did not elicit any emotions. However, in both years, the emotion that was reported significantly more frequently across different types of online sources was anger. In 2020, we observed a negative emotional response when the sources of information were the CPH or hospital websites, while in 2021, we found a significant increase in calmness and decline in anger related to the health information obtained from the CPH (Figure 2). Anxiety and anger were associated with online search for health information, particularly through social networks and news websites (Figure 2).

## Discussion

Between 2020 and 2021, we observed a change in the perceived reliability of information sources related to the COVID-19 pandemic, the frequency of search, and the channels of information search.

While in 2020 most respondents relied on the information provided by the CPH, this rate in 2021 decreased to less than half of the respondents, which is similar to the results observed in other studies (49,50). The findings from 2021 may be related to the respondents’ beliefs that the CPH provided less clear and less useful information, and that individuals from the CPH were no longer as reliable as in the first year. Furthermore, respondents in 2021 believed that the information provided by the CPH was based on daily politics. This finding may be relevant for public health, since trust in the government during the COVID-19 pandemic was correlated with the compliance to the government’s policy (51) and COVID-19 preventive measures (52).

Our respondents most frequently used online media and TV to obtain information about the COVID-19 pandemic, similar to respondents from other countries (38). In 2021, the number of respondents who used online media as an information source increased compared with the number of those who used TV. The explanations for this finding could be that daily press conferences by the CPH were no longer broadcast on TV and that people watched TV less often as the lockdown ended.

In 2020, more than half of the respondents reported the need to seek additional information about COVID-19 online, but this rate declined significantly in 2021. In 2020, respondents more frequently searched for the disease-related information as they did not know much about the disease. In contrast, in 2021, they might have become overloaded with COVID-19-related information. A recent study from the United States suggested that people may intentionally avoid information in order to avoid disagreements and confrontations with their social contacts (53). However, additional online information on COVID-19 was perceived as useful.

Our respondents thought that information acquired online was confusing and often contradictory. A study assessing COVID-19-related health literacy in Germany concluded that frequent use of social media websites may cause confusion (41). Furthermore, our participants agreed that searching for online information requires knowledge on the reliability and accuracy of the information. This belief was not significantly changed in 2021.

The reliability of all three media-exposed members of the CPH significantly declined in 2021. Our survey in 2020 was conducted after the end of the first lockdown in Croatia (54). At that time, the trust in the CPH might have been influenced by a low number of infections nationwide and the upcoming relaxation of COVID-19 measures. In 2021, besides all other negative social effects of the pandemic, all three assessed members of the CPH faced criticism by the media (55).

In 2020, a highly ranked government politician was perceived as having medium reliability, while another highly ranked politician, who was a medical doctor, was perceived as highly reliable. In this study in general, scientists and medical doctors were rated as more reliable than politicians, so we assume that the latter politician’s higher reliability was related to his medical profession, rather than to his politics. A survey that covered six countries also reported skepticism toward individual politicians, while health authorities and experts were highly trusted (56). Again, the reliability of both of politicians declined in 2021, just as the reliability of health professionals and scientists. Some scientists were unknown to most respondents, probably because they were underrepresented in the media.

The trust in governmental institutions regarding COVID-19 control correlated with higher adoption of health behaviors (57), and a study from 2020 showed adherence to the CPH measures (58). At that time, the CPH was considered a professional and knowledgeable body that provides clear and useful information, and measures in 2020 seemed to yield desirable outcomes (54). Furthermore, individuals from the CPH were mostly health professionals, and were rated as highly reliable in 2020.

The respondents pointed out the importance of accuracy and reliability of information from online sources. They highlighted scientific or professional soundness of information and trustworthiness of the information source over political influence, bias, and contradictory or outdated information. In 2021, respondents found the reliability of the pandemic-related information to be even more important. This may be due to the efforts of professionals who provided guidance to the general public about the reliability of health-related information (59).

In this study, negative emotional states, such as anxiety and anger, were associated with online search for health information, particularly through social networks and news websites. In a Finnish study, information anxiety was not correlated with obtaining information through mass media and other internet sources, but with an information overload from these sources (60).

Our study has several limitations. At the beginning of the pandemic there were no developed instruments on the study subject, especially in the Croatian context. Therefore, we developed our own instrument, for which we performed the face validity assessment and a very brief content validity assessment. We were not able to assess construct validity because our instrument was not developed in a way to measure a unidimensional construct with standardized and same form of items (61). Our intention was to collect a wide range of opinions related to the COVID-19 pandemic at two assessment points. Further validation should be performed in future studies. Our findings can be only partly transferred to other countries, since the obtained information, communication system, and the investigated period were specific to Croatia. Furthermore, our survey did not specify individual news websites but grouped all online media together. Finally, our sample consisted only of internet users, since the survey was distributed online. Although we used similar distribution channels, the study sample from 2020 was not the same as the sample from 2021. Since we needed to protect the participants’ anonymity, we could not register the IP addresses of participants and follow the same recipients in 2021. In addition, the sampling was performed with a non-probabilistic sampling method.

The study conclusions may be used to inform health communication in pandemic circumstances, especially for ongoing vaccination campaigns. First, the CPH, which may be used as a proxy for any central government body in charge of the pandemic management (62), was considered a popular, reliable source of information when they were perceived to be effectively managing the pandemic. Online media replaced TV when people stopped spending most of their time at home, so the online media should be considered for use when creating campaigns. One of the most valuable conclusions of our study was the increased awareness of the reliability of COVID-19-related information after a year of the pandemic. This phenomenon should be addressed in other countries as well.

## Additional Material

Supplementary Figure 1

Supplementary Msterial 1
