# Supplementary material for: Changes in information-seeking patterns and perception of health crisis management in a year of COVID-19 pandemic: a repeated cross-sectional study
Source: Croat Med J. 2023 Apr;64(2):93–102. doi: 10.3325/cmj.2023.64.93 (PMC10183963; doi:10.3325/cmj.2023.64.93)
Supplement: Supplementary Figure 1 [file CroatMedJ_64_s001.pdf]

Supplemental Figure 1

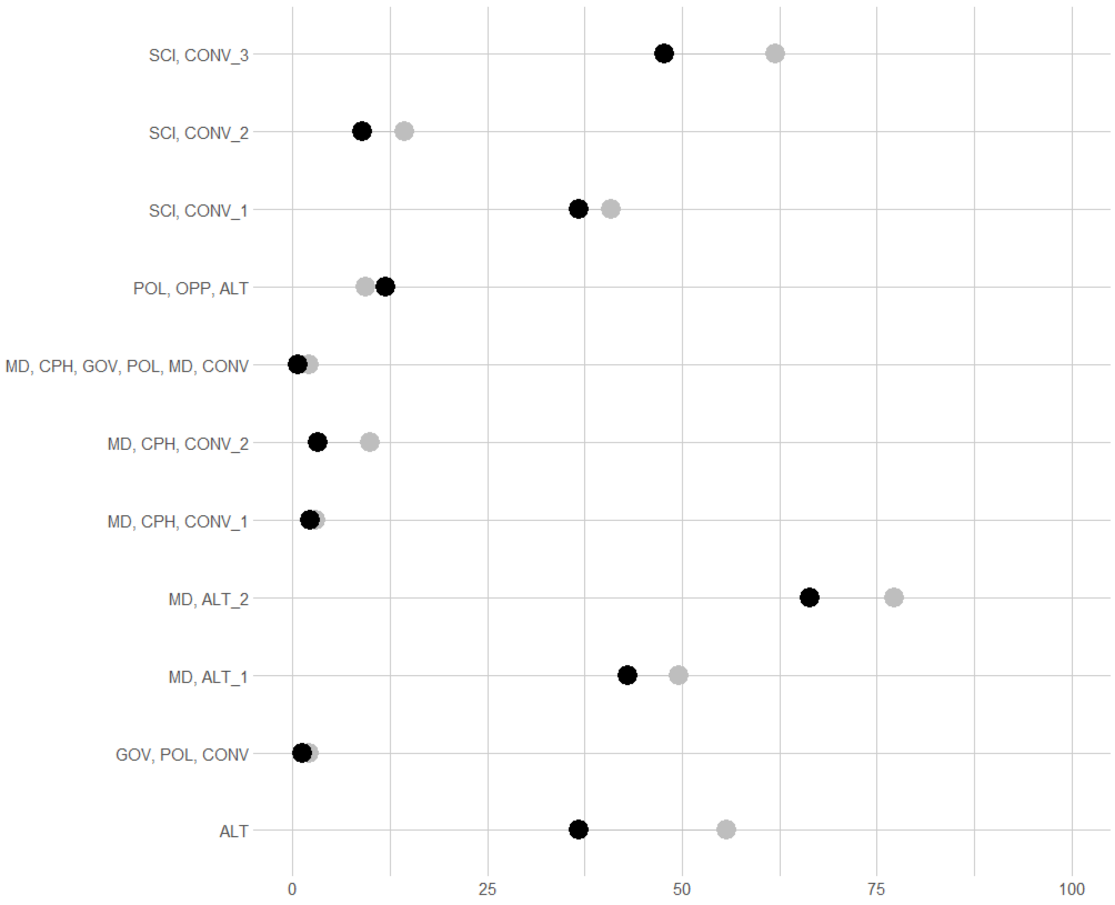

Proportion of participants from 2020 (black dot) and 2021 (grey dot) who stated that they are not familiar with specific public person[1]

[1] Individual public figures are assigned with a codes in accordance with their relevant characteristics: GOV - an individual was part of the government, a member of political party with a majority in parliament; SCI – a scientist; MD – a medical doctor; CPH – a member of Civil Protection Headquarters; POL – a politician; OPP – a person was a member of parliamentary opposition party; CONV – a person who promoted conventional attitudes medicine and public health; ALT – a person who promoted alternative, pseudo-, or non-scientific attitudes in medicine and public health.
