# Supplementary material for: Changes in information-seeking patterns and perception of health crisis management in a year of COVID-19 pandemic: a repeated cross-sectional study
Source: Croat Med J. 2023 Apr;64(2):93–102. doi: 10.3325/cmj.2023.64.93 (PMC10183963; doi:10.3325/cmj.2023.64.93)
Supplement: Supplementary Msterial 1 [file CroatMedJ_64_s002.pdf]

Supplementary Questionnaire

Questionnaire about use of information during COVID-19 pandemic outbreak

1. Sex: a. male b. female c. I do not want to declare

2. Year of birth: _______________

3 What is your current highest level of school completed/degree received?

a. without elementary school education

b. elementary school

c. secondary school graduate

d. polytechnic degree/university undergraduate degree (*VŠS* – ‘’higher expertise’’, t/n[1]) or bachelor’s degree (bachelor)

e. university graduate (VSS – ‘’higher expertise’’, t/n) or master’s degree (master)

f. master of science (MSc) or a degree in specialist postgraduate studies (univ. spec.)

g. doctorate degree (PhD)

4. What is your working status?

a. Employed

b. Unemployed

c. Student

d. Retired

e. Other. Please enter here ________________

5. Do you belong to a category of a physician or any other healthcare worker?

a. YES b. NO

6. Please describe your health condition:

a. I do not have serious health issues

b. My health condition includes (it is possible to choose more than one option)**:**

[1] t/n – translator's note

- chronic pulmonary diseases (chronic obstructive pulmonary disease, emphysema, chronic bronchitis, pulmonary fibrosis)

- moderate or severe asthma

- cardiovascular diseases (heart failure, narrowing of the arteries, congenital heart disease, pulmonary hypertension, cardiomyopathy)

- diabetes (type 1 or type 2)

- weak immune system (due to tumour treatment, corticosteroids treatment, organ transplantation, immunodeficiency, immune system diseases or similar)

- kidney disease (dialysis treatment)

- overweight

- liver diseases (cirrhosis)

7. Please, rate your health condition with a grade 1 – 5, where 1 corresponds to a weak health condition, and 5 corresponds to an excellent health condition.

____

8. Were you more concerned for your health during COVID-19 pandemic than usual?

a. YES b. NO

9. Please indicate your level of agreement/disagreement on each of the following statements:

	**1** Strongly disagree	**2** Disagree	**3** Neither agree nor disagree	**4** Agree	**5** Strongly agree
Coronavirus presents serious health issue for me.					
I am afraid that I could get infected with coronavirus.					
I will probably get infected with coronavirus and get sick.					
I feel that the contagion would be very dangerous for me.					

10. During COVID 19 pandemic I mostly relied on information provided by Civil Protection Headquarters:

a. YES b. NO

11. The medium I used most frequently for informing about COVID-19 pandemic was:

a. television

b. radio

c. online media

d. printed media

e. I did not follow media

12. I had the urge to seek additional information on COVID-19 online:

a. YES b. NO

13. I had the urge seek additional information about COVID-19:

a. Less than once a day

b. Only once a day

c. Two times a day

d. Three or more times a day

14. Please indicate your level of agreement/disagreement on each of the following statements:

	**1** Strongly disagree	**2** Disagree	**3** Neither agree nor disagree	**4** Agree	**5** Strongly agree	Not applicable
Information provided by Civil Protection Headquarters were clear and useful.						
Civil Protection Headquarters consists of professionals who know what they are doing, and acts based on knowledge.						

Information given by Civil Protection Headquarters are result of politics and not medical profession.						
Articles on pandemic in daily print are sensationalistic.						
Journalists are well informed on pandemic.						
Newspaper articles are politically biased.						
Information on pandemic acquired online are confusing and often contradictory.						
Information on pandemic acquired online are useful when we want to be informed more.						
When searching information online one should watch for accuracy and reliability of information.						

15. Please choose one of the options for each source of online health information.

**When I searched online health information…**					
	I felt calm	I felt anxiety	I felt anger	I did not feel any emotions	I did not search
From Civil Protection Headquarters					
Through Internet news portals					
Through Social networks					
Through forums					
Through YouTube videos					
On hospital websites					

16. Please choose one of the following options for each statement.

When I read information in media about COVID-19 I wonder:					
	**1** never	**2** rarely	**3** sometimes	**4** very often	**5** always
..if the information is under political influence.					
…if the content is scientifically or professionally sound.					
…whether the source is trustworthy of fake.					
…if the information is up to date or outdated.					
…whether the source is independent, or the data depends on other sources.					
…if the information collected from the source is biased.					
…if there are contradictory information.					

17. How would you rate reliability of the following persons[2] as a source of information related to COVID – 19 (1- completely unreliable, 5 – absolutely reliable, N – I do not know who the person is):

a. MD, CPH, GOV, POL, MD, CONV ___
b. MD, CPH, CONV_a ___
c. MD, ALT_a ___
d. SCI, CONV_b ___
e. ALT ___
f. SCI, CONV_a ___
g. SCI, CONV_c ___
h. POL, OPP, ALT ___
i. MD, ALT_b ___

[2] In order to avoid the use of personal names in the published version of the questionnaire, we coded the individual public figures by assigning the short codes to each person, in accordance with their characteristics relevant for the research, as follows: GOV - an individual was part of the government, a member of political party with a majority in parliament; SCI – a scientist; MD – a medical doctor; CPH – a member of Civil Protection Headquarters; POL – a politician; OPP – a person was a member of a parlamentary opposition party; CONV – a person who promoted conventional attitudes in medicine and public health; ALT – a person who promoted alternative, pseudo-, or non-scientific attitudes in medicine and public health. One public fugure may be assigned with several codes in accordance with his/her characteristics.

j. MD, CPH, CONV_b ___
k. GOV, POL, CONV ___

Thank you for your participation!
